# Monitoring the Temporal Expression of Genes Involved in Ochratoxin A Production of *Aspergillus carbonarius* under the Influence of Temperature and Water Activity

**DOI:** 10.3390/toxins9100296

**Published:** 2017-09-22

**Authors:** Iliada K. Lappa, Dimosthenis Kizis, Efstathios Z. Panagou

**Affiliations:** 1 Laboratory of Microbiology and Biotechnology of Foods, Department of Food Science and Human Nutrition, Agricultural University of Athens, Iera Odos 75, 11855 Athens, Greece; l.lappa@aua.gr (I.K.L.); d.kizis@bpi.gr (D.K.); 2 Laboratory of Mycology, Department of Phytopathology, Benaki Phytopathological Institute (BPI), St. Delta 8, 14561 Athens, Greece

**Keywords:** *Aspergillus carbonarius*, OTA, biosynthetic genes, temperature, water activity, SGM

## Abstract

The objective of this study was to investigate the effect of environmental factors, namely temperature and water activity, on genes involved in the regulation of ochratoxin A (OTA) production over time. For this purpose, the previously characterized toxigenic *Aspergillus* *carbonarius* Ac29 isolate from Greek vineyards and the *A. carbonarius* ITEM 5010 reference strain were subjected to combined temperature and water activity (a_w_) treatments to study OTA production and relative gene expression. The fungal isolates were grown on a synthetic grape juice liquid medium (SGM) under different temperature (20 °C, 25 °C and 30 °C) and a_w_ (0.94 and 0.98) regimes. The expression of the AcOTA*pks*, AcOTA*nrps*, and *laeA* OTA related genes was investigated using real time PCR. Gene expression was monitored at the same time points, along with fungal biomass and OTA accumulation at three, six and nine days of incubation. In gene expression analysis, stimulation of the biosynthetic genes was observed a few days before any toxin could be detected. This fact may underline a possible early indicator of potential toxin contamination of grapes. However, the transcript levels varied with respect to the different combinations of ecophysiological conditions and time, highlighting a complex regulation of OTA related gene expression of *A. carbonarius* in the specific medium.

## 1. Introduction

Ochratoxin A (OTA) is the most important mycotoxin encountered in grapes and grape products, which is widely distributed as a natural contaminant. It is categorized as group 2B carcinogen by the World Health Organization (WHO) [1] as it displays nephrotoxic, hepatotoxic, teratogenic and immunosuppressive properties affecting seriously human health. In general, ochratoxins (OTs) are secondary metabolites produced by several species of filamentous fungi belonging to the *Aspergillus* or *Penicillium* genera. *Aspergillus carbonarius* is considered the most important OTA producer in grapes, especially for those cultivated in warm climates [2].

The basic chemical structure of OTs, concerning OTA, OTB, and OTC, consists of an isocumarin nucleus bonded to a l-phenylalanine unit by an amide bond. OTA is the chlorinated form of the toxin, which is most prevalent, whereas B and C, the non-chlorinated and esterified forms, respectively [3]. Its structure indicates that enzymatic reactions are needed for metabolite biosynthesis. Even though this part has not been fully explored, a number of putative pathways have been recently revealed and genes related to OTA biosynthetic pathway and regulations have been suggested.

More specifically, according to OTA molecular structure and proposed biosynthetic pathways, its synthesis requires several proteins, including a polyketide dihydroisocoumarin synthase (PKS), a non-ribosomal peptide synthase (NRPS) for ligation of the amino acid phenylalanine and the polyketide, and a halogenase for chlorination [4]. As Hertweket [5] reported, most of the known mycotoxins produced by fungi consist of a polyketide or peptide catalyzed by PKS or NRPS large multimodular enzymes. The functional role of *pks* and *nrps* genes has been revealed in studies concerning different fungal genera such as *Penicillium nordicum*, *A*. *carbonarius*, *A*. *ochraceus*, *A*. *westerdijkiae* and they have been established to date as OTA biosynthetic key enzymes [6,7]. Since both genes have been demonstrated to be necessary in toxin biosynthesis, they have been used as targets to detect and quantify OTA producing molds by molecular techniques. Furthermore, among mycotoxin gene clusters, regulatory genes also exist to control the expression of these biosynthetic genes. LaeA is a protein known as secondary metabolite regulator of various filamentous fungi, being also involved in the regulatory pathway for OTA biosynthesis as a global transcriptional factor [8].

The presence of OTA in food products could be the result of direct contamination by molds and its accumulation may be affected by different factors such as temperature, a_w_, pH and substrate composition such as carbon and nitrogen sources [9]. From the food safety perspective, evaluation of induction of these key genes could become an indicator tool for risk assessment of toxigenic species since gene transcription usually precedes phenotypic production.

The purpose of this study was to employ reverse transcription quantitative PCR (RT-qPCR) to explore the influence of temperature, a_w_ and time on OTA related gene transcripts expression pattern. While plenty of data exist on the effect of the above factors on growth and OTA production of *Aspergillus carbonarius*, there are no data on the impact of these factors at gene expression level. Το our knowledge this work is the first attempt to investigate the effect of ecophysiological factors such as temperature and a_w_ on *Aspergillus carbonarius* gene expression along with OTA production in relation to time.

## 2. Results and Discussion

### 2.1. Impact of Ecophysiological Factors on Growth and Toxin Production over Time

**Figure 1:**
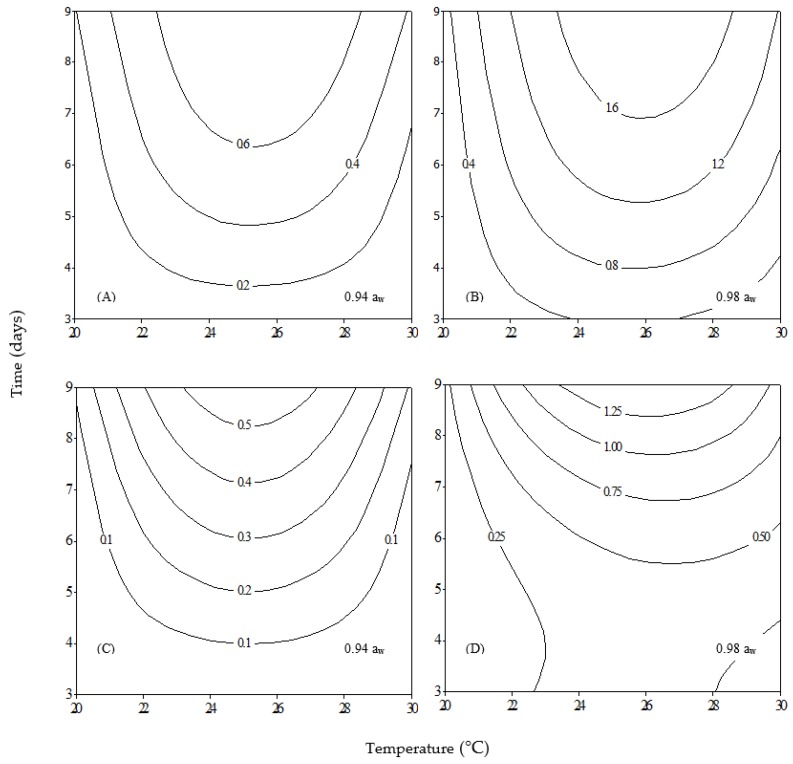
Two-dimensional contour plots showing the effect of temperature and incubation time on biomass production (g) for: *Aspergillus carbonarius* Ac29 (**A**,**B**); and *A*. *carbonarius* 5010 (**C**,**D**).

Two-dimensional (2D) contour plots representing biomass production (g) of *A. carbonarius* Ac29 and *A. carbonarius* ITEM 5010 in relation to temperature and incubation time for each a_w_ level assayed are shown in Figure 1 (biomass and OTA raw data are provided in Supplementary Materials). Higher biomass was produced at 0.98 a_w_ (Figure 1B,D) for both fungi irrespective of temperature and incubation time compared to 0.94 a_w_ (Figure 1A,C).

Moreover, an intra-strain difference was observed since the Ac29 isolate produced more biomass compared to the reference strain ITEM 5010 in all combinations of temperature × time for the same a_w_ level. In general, biomass production increased proportionally with time for both fungi, and reached a maximum at 25 °C and 0.94 a_w_, whereas for 0.98 a_w_ maximum biomass production has shifted to 26 °C. It needs to be noted that at day 3 no growth was observed for both strains at 0.94 a_w_ regardless of temperature and at 0.98 a_w_ at 20 °C. The above results are consistent with other ecophysiological studies for *A. carbonarius* regarding optimum growth temperature [10,11]. These results indicate that the combination of specific temperature and water activity has an important effect on fungal growth. There are reports investigating a number of *A. carbonarius* isolates, showing that low a_w_ (ca. 0.94) is restrictive not only to growth, but also to OTA production, especially when they act in parallel with other environmental parameters [12].

**Figure 2:**
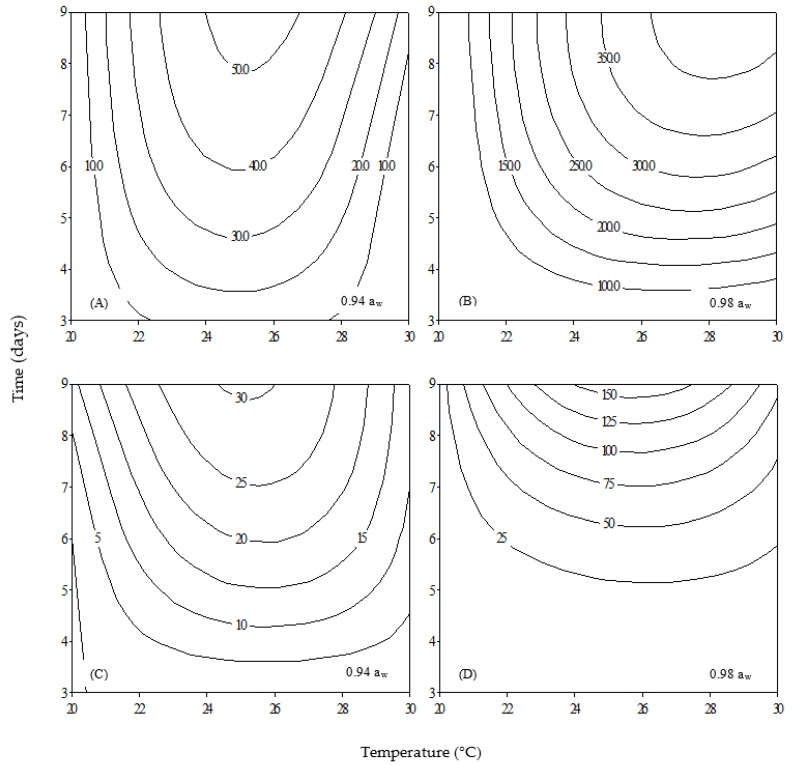
Two-dimensional contour plots showing the effect of temperature and incubation time on OTA production (ng/mL) for: *Aspergillus carbonarius* Ac29 (**A**,**B**); and *A. carbonarius* 5010 (**C**,**D**).

Figure 2 presents 2D contour plots for OTA production of the two fungal strains in relation to temperature and time for each a_w_ level separately. OTA accumulation levels varied reaching a maximum value of 423 ng/mL, with the isolate Ac29 being a higher OTA producer compared to the reference strain ITEM 5010, enforcing thus the finding of a previous study in which the selected fungal strains present high OTA potential [13]. In general, a similar trend was observed in both strains as OTA gradually increased throughout incubation time. It has already been reported [14] that reduction of a_w_ in grapes results in reduction of OTA accumulation. On the other hand, high a_w_ levels (in the range of 0.98) enhance OTA production as reported by other researchers [15,16]. The optimum temperature for OTA production was different for the two strains at 0.98 a_w_, since Ac29 presented the highest toxin concentration at 28–30 °C in contrast to ITEM 5010 where maximum toxin accumulation was observed at ca. 26 °C (Figure 2B,D). However, at 0.94 a_w_, both fungi presented maximum OTA production at ca. 25–26 °C (Figure 2A,C).

Our results are consistent with previous studies which have reported 25 °C as optimum temperature for OTA production by *A. carbonarius* [15], and also agree with Belli et al. [17] who reported 30 °C as the temperature of maximum OTA production observed. However, other studies report 20 °C as the optimum temperature for toxin production [18,19]. Such differences are possibly associated with the use of different isolates [20], growth media [21] and experimental conditions such as solid or liquid growth cultures [22]. Furthermore, even thought it has been demonstrated previously that toxin production is well associated with biomass for both strains [13], the fact that Ac29 produced higher amounts of OTA at 30 °C could suggest that this strain is adapted to high temperatures, since it has been isolated from vineyards in Crete, a warm climate region in Southern Greece.

Finally, it needs to be noted that isolate Ac29 did not produce any OTA at 20 °C and 30 °C/0.94 a_w_, whereas no toxin was detected for strain ITEM 5010 at 20 °C/0.94 a_w._ In the cases where no toxin was detected, growth was macroscopically visible, pointing that ecophysiological conditions that allow fungal growth are broader than those required for OTA production [23]. This highlights the significance of the combination of the above ecological factors on toxin production and also the importance of intra strain studies. Furthermore, analysis of variance performed on OTA revealed highly statistical significance (*p* < 0.0001) for all single factors assayed (strain, temperature, a_w_ and time) (Table 1). Among the parameters tested, a_w_ had the strongest effect followed by strain, time and finally temperature. Moreover, ANOVA assessed the impact of cross effect among the parameters assayed and the combination of strain x a_w_ presented the highest impact. Furthermore, comparison of the mean values for OTA revealed differences between the two strains at each incubation time at 25 °C and 30 °C/0.98 a_w_ which is also graphically confirmed by the 2D contour plots (Figure 2B,D). In addition statistically significant differences were observed between the two strains at 20 °C/0.94 a_w_ after nine days of incubation. From the intra-species perspective, temperature had the same impact on both strains in terms of toxin production. However, a_w_ had greater impact on strain Ac29 (*p* < 0.0001) in contrast to reference strain (*p* < 0.0004) which appeared to be more sensitive to the parameter of time.

### 2.2. Impact of Ecophysiological Factors upon Gene Regulation over Time

**Figure 3:**
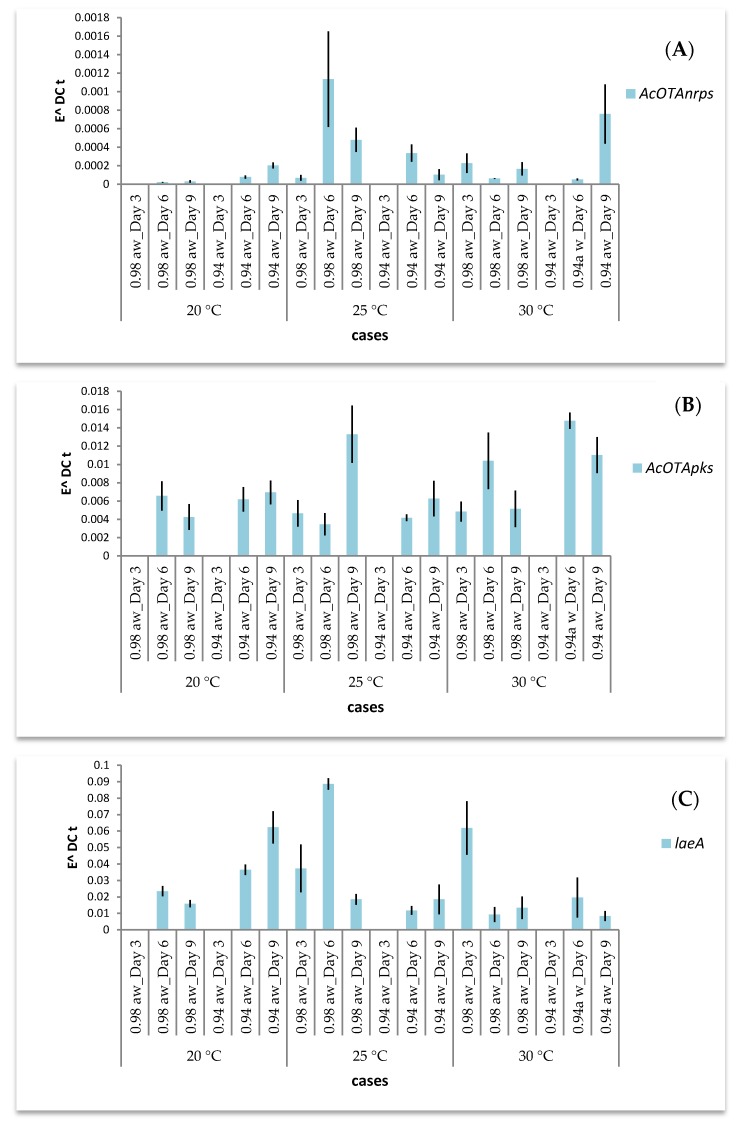
Effect of temperature and a_w_ on transcript levels of: AcOTA*nrps* (**A**); AcOTA*pks* (**B**); and *laeA* (**C**) genes of *Aspergillus carbonarius* Ac29 through time. Error bars indicate standard error of three replicates. In the case of absence of fungal growth at Day 3, no values are displayed.

**Figure 4:**
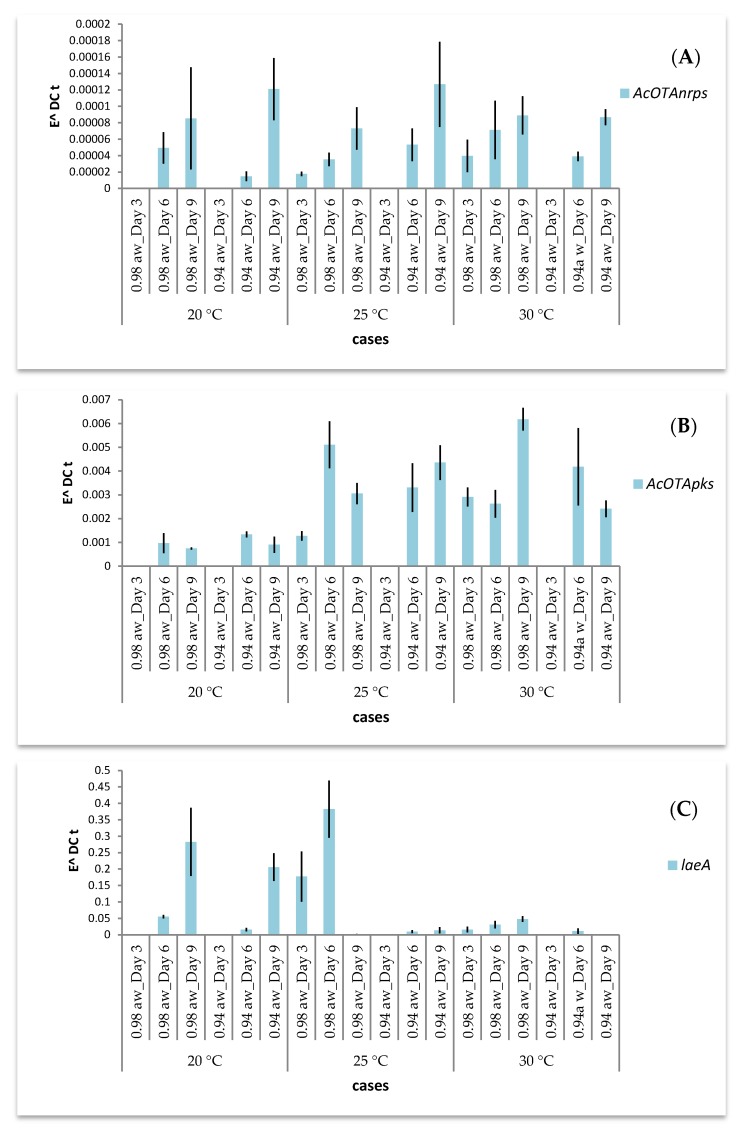
Effect of temperature and a_w_ on transcript levels of: AcOTA*nrps* (**A**); AcOTA*pks* (**B**); and *laeA* (**C**) of reference strain *Aspergillus carbonarius* ITEM 5010 through time. Error bars indicate standard error of three replicates. In the case of absence of fungal growth at Day 3, no values are displayed.

The effect of incubation temperature, a_w_ and time on the expression of AcOTA*nrps*, AcOTA*pks* and *laeA* genes during *A. carbonarius* growth in SGM are presented in Figure 3 and Figure 4 for Ac29 and ITEM 5010, respectively. In general, with regard to the expression of the two biosynthetic genes and the global regulator, both strains showed regulation in all cases studied and different expression patterns were observed. Statistical analysis showed that gene expression of all three genes was significantly affected by the parameters used in the study (Table 2A). Analysis of variance for the whole response of the selected genes revealed high statistical significance for every single factor (Table 2B). The effect of temperature on gene expression was analyzed at different incubation times. A *t*-test revealed that AcOTA*pks* (*p* < 0.0225) and *laeA* (*p* < 0.0328) were the genes mostly affected by the different temperature regimes, especially at Day 6 where the highest gene expression was observed. 

Moreover, a_w_ effects were evaluated at different sampling times indicating that gene expression at Day 6 was highly affected by a_w_ levels. Specifically, AcOTA*nrps* expression of Ac29 was higher at 25 °C/Day 6 at both a_w_ levels assayed. In addition, at 20 °C and 30 °C, gene expression levels for the same fungus increased with time at 0.94 a_w_ (Figure 3A). AcOTA*pks* expression did not present any difference at 20 °C, but up-regulation was observed at 25 °C and 30 °C (Figure 3B). The levels of *laeA* expression increased from 20 to 25 °C followed by a decrease at 30 °C (Figure 3C). On the other hand, the general trend of AcOTA*nrps* expression for the strain 5010 followed an increase with temperature and the same holds for AcOTA*pks* transcripts (Figure 4A,B). Finally, the expression of *laeA* gene decreased with increasing temperature (Figure 4C).

Differences among transcript levels were also observed at different sampling times when *A. carbonarius* grew at constant temperature. Increase of incubation time resulted in a strong trend of turning up transcription of AcOTA*nrps* for strain ITEM 5010 at all three temperatures assayed and for Ac29 at 20 °C and 30 °C. Furthermore, for the reference strain, expression of *laeA* followed an increasing trend with time at 20 °C and 30 °C/0.98 a_w_ (Figure 4C). Hence, sampling time caused greater transcriptional changes than growing conditions. The influence of time on AcOTA*pks* at different sampling times did not provide a clear trend in both fungi.

According to Table 2A, fungal strain caused the highest variation in gene response for the ecophysiological parameters studied. Moreover, results showed that AcOTA*pks* (*p* < 0.0001) gene was most highly affected by fungal strain. Further, analysis of interactions by ANOVA showed the impact of the combined effect of the ecophysiological parameters studied on gene expression at intra-strain level (Table 2B). The highest impact is presented for each strain underlying the complexity of interactions of all parameters on gene expression results.

**Figure 5:**
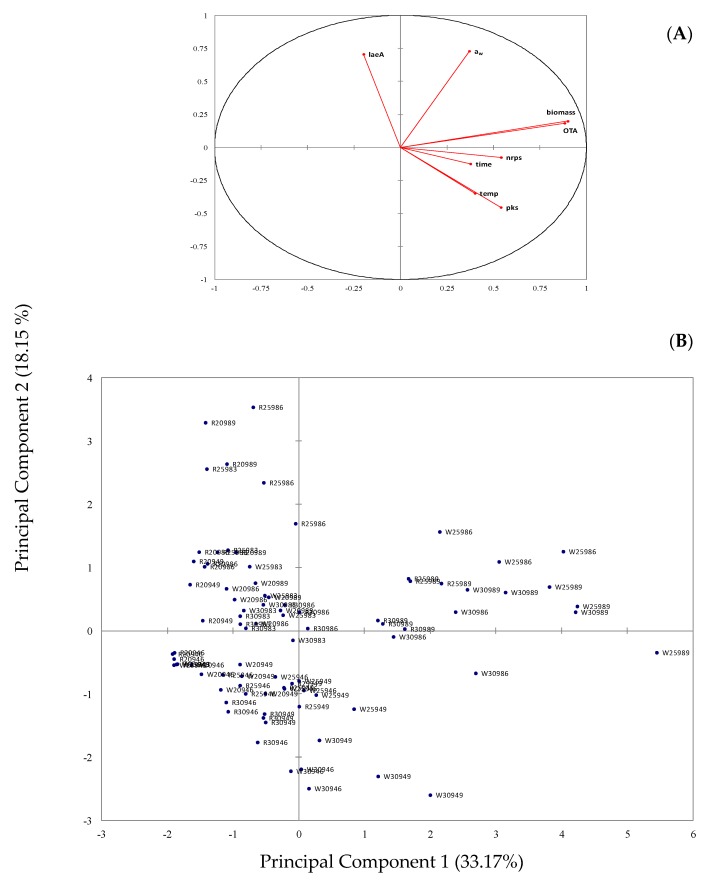
Plot of: variables (**A**); and cases (**B**) of the principal components analysis illustrating the correlation between a_w_, temperature, fungal biomass, OTA production and temporal expression of *Aspergillus carbonarius* OTA related genes by Ac29 and reference strain ITEM 5010, grown on SGM. (Cases are coded as follows: W and R correspond to wild and reference fungal strain, respectively; the following two numbers correspond to incubation temperature; the next two digits indicate a_w_ level; and the last digit incubation time).

Principal Components Analysis (PCA) undertaken on the samples corresponding to different combinations of temperature, a_w_, time, and fungal strain resulted in the identification of three Principal Components (PCs) with eigenvalues higher than 1.0 which explained 67.3% of the total variance. Moreover, the results of the analysis are graphically illustrated in Figure 5 where the original variables are projected onto the plane formed by the selected two first PCs.

Specifically, Figure 5A can be used to establish relationships among variables. Thus, a strong positive correlation was observed for OTA and biomass production, indicating that the higher the biomass the higher the amount of OTA [13]. A close relationship was also evident for temperature and AcOTA*pks* and AcOTA*nrps* gene expression, indicating that higher temperatures resulted in higher expression of these genes, whereas the opposite was observed for temperature and *laeA* gene expression. According to this graph, there is no relationship between OTA and *laeA* gene expression as the two vectors corresponding to these variables form an angle close to 90 °C [24]. It is also notable that the vectors for AcOTA*nrps* and AcOTA*pks* gene expression are located in diagonally opposed quadrants in relation to *laeA* gene expression, meaning that, when the former two genes are up regulated, the latter gene is down regulated. Finally, the vector for time is correlated to AcOTA*nrps*, and AcOTA*pks* genes, indicating higher expression of these genes with the course of time. 

The distribution of the samples on the plane formed by the first two PCs is illustrated in Figure 5B. The wild strain Ac29 is mainly located in the right part of the plot, especially for the cases characterized by high a_w_ (0.98) and high incubation time (nine days) at 25 and 30 °C. Moreover, by taking into account the plot of variables (Figure 5A), it is clear that the fungus Ac29 grown at these conditions is associated with higher biomass and OTA production and higher expression of the AcOTA*pks* and AcOTA*nrps* genes. The remaining cases for Ac29 corresponding to low a_w_ (0.94) regardless of temperature and time as well as those cases associated with 20 °C irrespective of a_w_ and time are located in the left part of the plot together with the reference strain without presenting a clear pattern. Certain cases of the ITEM 5010 located in the upper left quadrant of the plot, corresponding to high a_w_ (0.98), 20 °C and 25 °C and diverse incubation times, are associated with the expression of the *laeA* gene that seems to present high expression at these conditions.

As reported above, AcOTA*nrps*, AcOTA*pks* and *laeA* have been systematically correlated with OTA production. However, it should be highlighted that there are no previous studies investigating the combined effect of different temperature regimes, a_w_ levels and time on the transcriptional responses of *A. carbonarius*. In the current work, the focus was given on two structural genes localized in the ochratoxigenic biosynthetic cluster, and one global regulator. In general, there are limited studies elucidating the expression of toxigenic related genes with different ecophysiological parameters. Specifically, the association between temperature, a_w_ and transcript profiles concerning mycotoxin production has already been published [25,26]. In the present work, even though a_w_ was the key factor affecting OTA production, this was not observed with expression patterns, where temperature seemed to act as the key factor influencing transcript levels. These results are in accordance with Yu et al. [27] who reported that temperature is a modulator of mycotoxin production, such as aflatoxins, indicating that high temperature negatively affects aflatoxin production by turning down transcription of the two key transcriptional regulators. However, mixed responses of different regulation between evaluated parameters occurred elsewhere. Recently, Gallo et al. [28] also observed that temperature was correlated to the induction of expression of structural biosynthesis genes, but not to that of aflatoxin regulatory genes. Rocha et al. [29] also reported weak association between fumonisin production by *Fusarium verticillioides* and some of FUM genes expression levels. In another work [30], it was found that FUM2 and FUM21 gene expression levels were slightly affected by modification of a_w_ whereas temperature was the main controlling factor in fumonisin B_1_ production by two strains of *Fusarium verticillioides*. Furthermore, the profiles of gene expression of both toxigenic strains analyzed were quite different. These differences on gene regulation indicate the importance of strain variability in these experiments, since the majority of studies usually investigate the behavior of one fungal strain.

The regulatory mechanism underlying OΤA biosynthesis is not completely understood yet, possibly due to its complexity through different levels of regulation. This mechanism could act within the biosynthetic cluster or external to it and its phenotypic expression is likely to subordinate to other regulatory processes acting at post-transcriptional level. It is also notable that translational or post translational control may also occur in a co-regulation mechanism of the genes studied [31]. Growth conditions that favor the expression of OTA biosynthetic genes do not always result in OTA biosynthesis by fungi. This could suggest that the influence of abiotic factors is mediated via induction of transcription of ochratoxin A biosynthetic genes. In fact, production of fungal secondary metabolites could be affected by numerous signals of inhibitory or promotional effect on the regulatory systems. Patterns in transcript biosynthesis are not necessarily correlated with phenotypic metabolites produced.

Finally, gene expression was assayed by RT-qPCR in relation to abiotic factors known to influence ochratoxin A biosynthesis. Molecular measurements are more sensitive compared to the analytic quantification of OTA, indicating the successful implementation of analysis of transcriptome response. Black aspergilli are normally present in vineyards, but OTA is not always detected even though it can be quantified at very low concentrations [32]. In this regard, RT-qPCR provides very useful information to relate molecular changes under different ecological conditions in a rapid and convenient way.

## 3. Conclusions

The influence of the environmental factors of temperature and a_w_ has been studied in this work and different profiles were observed in response to time concerning OTA and gene expression levels by *Aspergillus carbonarius.* The expression of OTA key biosynthetic genes during growth on SGM at different environmental conditions was successfully assessed, since transcripts were detected in all cases. Early activation of both OTA biosynthetic key genes confirmed the predictive nature of RT-qPCR analysis, since molecular indicator genes were expressed a few days before any OTA could be detected. High variation between the wild fungal isolate Ac29 and the reference strain ITEM 5010 has been observed underlying the significance of intra-species variability. Temperature and a_w_ were involved in the transcription process of the specific genes for mycotoxin production. Stimulation of genes and the observed transcript levels may suggest a different regulation action evolved. This indicates that expression is possibly closely related with control at the post-transcriptional level and requires further investigation. Even though OTA and biomass appeared to have a constant trend with the environmental parameters under study, a determined pattern could not be clearly established between transcript levels and toxin production. Results constitute a first indication on the responses in different ecophysiological environments, highlighting the complexity of regulation mechanisms at gene expression level of *A. carbonarius* and suggesting further research on the fine regulation of gene expression.

## 4. Materials and Methods

### 4.1. Fungal Isolates and Culture Media

A wild isolate of *A. carbonarius* from grapes of Greek vineyards was used throughout this study. The isolate, coded Ac29, has been previously characterized by molecular methods [33] and belongs to the fungal culture collection of the Laboratory of Food Microbiology and Biotechnology (LFMB) of the Agricultural University of Athens (AUA). In addition, *A. carbonarius* ITEM 5010 was kindly provided by Prof. Tsitsigiannis from the AUA Phytopathology Department and used as reference strain. It is a genome sequencing ochratoxigenic strain of *A. carbonarius*, isolated from grape berries (Apulia, Italy) and it has been already used as reference strain in similar gene expression studies [34,35].

Spore suspensions of each fungus were prepared using 7-day-old colonies grown on Malt Extract Agar at 25 °C. Conidia were harvested from sub-cultures in an aqueous solution of 0.01% Tween 80 (Merck, Schuchardt, Hohenbrunn, Germany) by scraping the surface of the mycelium with a sterile glass rod. The concentration of the final spore suspension was assessed using a haemocytometer (Brand, Wertheim, Germany) and adjusted by appropriate dilutions to approximately 10^6^ spores/mL.

The study was performed on a Synthetic Grape Juice Liquid Medium (SGM) with pH adjusted to 3.8 using 2M KOH, according to Lappa et al. [13]. SGM was used in Erlenmeyer flasks containing 50 mL of medium inoculated with fungal spore suspension to provide an initial count of 10^6^ conidia/mL in the flask. The a_w_ of this basal medium was 0.98, as measured by an AquaLab LITE (Decagon Devices Inc., Pullman, WA, USA) water activity meter at 25 °C, and modified to the required level of 0.94 by adding glycerol [36]. Incubation was carried out at 20 °C, 25 °C and 30 °C for 9 days in a rotating shaker at 100 rpm. Rotation helped mycelia to be submerged into the liquid medium and suppress sporulation in order to avoid melanin production which would disturb RNA extraction procedure [22].

### 4.2. OTA Determination and Growth

For toxin determination, the whole content of each flask was analyzed at Days 3, 6 and 9 as reported elsewhere [22]. Specifically, each sample of 50 mL was homogenized using an Ultra Turax homogenizer (Heidolph Instruments, Schwabach, Germany) for 1 min at the highest speed. Subsequently, 5 mL of each homogenate were mixed with 5 mL of 100% methanol and left still for 30 min for OTA extraction. Furthermore, extracts were filtered through a Whatman No 2 filter paper in order to remove any suspended solids and filtered again through a 0.2 μm syringe-driven filter unit (Millex, Millipore Co., Bedford, MA, USA) All extracts were stored at −20 °C until analysis. OTA was determined by HPLC analysis as detailed elsewhere [13]. Briefly, a Model PU-980 Intelligent pump and an FP-2020 Plus fluorescent detector (JASCO Inc., Easton, PA, USA) were used. The analysis was performed under isocratic conditions at a flow rate of 1 mL/min of the mobile phase (water/acetonitrile/acetic acid; 49.5/49.5/1) through a Waters spherisorb C18 analytical column, 5μm ODS2 (4.6 × 250 mm) (Resteck Co., Pinnacle II, Bellefonte, PA, USA). Injection volume was 10 μL and run time for samples was 20 min with OTA detected at about 11 min. Additionally, known concentrations of OTA were spiked on SGM and a recovery assay for the HPLC method was assessed [22]. The detection limit of the analysis was 1 ng/mL of the measurement solution. For growth determination, fungal mycelium was harvested from the flasks, and biomass wet weight was determined at Days 3, 6, and 9. Both toxin and growth determination were conducted in triplicate.

### 4.3. RNA Isolation and cDNA Synthesis

RNA was extracted as described by Lappa et al. [22]. Briefly, 3 biological samples of grown mycelia were collected from SGM, washed with double distilled H_2_O (ddH_2_O), dried in Whatman No 2 paper, flash frozen in liquid nitrogen for stabilization of expression, and stored at −80 °C until extraction. Frozen tissues were lyophilized and approximately 10 mg of fungal mycelium were grounded to powder and used for nucleic acid extraction. Invitrogen, PureLink RNA mini kit (Ambion, Carlsbad, CA, USA) was used for total RNA isolation according to manufacturer’s protocol and Trizol (Ambion, Carlsbad, CA, USA) was employed in RNA extraction. Moreover, to remove genomic DNA contamination, samples were treated with Turbo DNase (Ambion, Carlsbad, CA, USA) according to kit instructions. The quality of RNA samples was checked through an RT-qPCR amplification to ensure absence of genomic DNA. RNA concentration and purity of each sample were assessed spectophotometricaly using a NanoDrop spectrophotometer (IMPLEN GmbH, Munchen, Germany). RNA integrity was also verified on 3% agarose ethidium–bromide staining gel.

First-strand cDNA was synthesized using PrimeScript Reverse Transcriptase (Takara, Kukatsu, Japan) according to manufacturer’s instructions. A concentration of 500 ng of total RNA was used for each sample in a 20 μL final reaction volume. Reaction mixture was incubated for 5 min at 65 °C and the specific set up program was maintained at 30 °C for 10 min, 42 °C for 60 min, and 70 °C for 15 min.

### 4.4. Real Time PCR

For gene expression assay, Real Time PCR was used to amplify the *Ac*OTA*pks*, *Ac*OTA*nrps* and *laeA* target genes. Constitutively expressed β-*tubulin* gene served as internal reference for gene expression normalization as this is a widely used housekeeping gene reported in relevant studies [4,8]. Nucleotide sequences of primers used in the qPCR assays are shown in Table 3.

The Applied Biosystems 7500 Fast Real-Time PCR System (Applied Biosystems, Foster City, CA, USA) was used to carry out Real time PCR experiments which were conducted in 96-well plates. To monitor cDNA amplification, qPCRBioSyGreen Mix High-Rox (PCR BIOSYSTEMS, London, UK) was used in a 10 μL reaction. Cycling conditions according to the specific SYBRGreen protocol were: 95 °C for 0.5 min, 60 °C for 0.25 min, and 72 °C for 0.25 min (40 cycles). Melting curve analysis of the PCR products was performed by heating to 95 °C for 0.15 s and 60 °C for 1 min, and continuous measurement of the fluorescence to verify the PCR product. Template-free negative controls were also used at every run. Data analysis was assessed by StepOnePlus RT-PCR System Software v2.1 and transcription levels of the target genes were determined after normalization with the reference gene (tub-β). In all cases, transcript values were expressed as normalized individual data points, based on the formula E^Δ*C*t^ [37], according to each primer pair efficiency, where Δ*C*_t_ = *C*_t_ gene of interest −*C*_t_ internal control. Gene expression measurements were considered to be comparable since equal amounts of RNA were used as template and also reaction conditions were the same in all qPCR assays. Gene expression was assayed in triplicate for each individual biological sample.

### 4.5. Data Analysis

Data concerning toxin production and gene expression levels were analyzed to investigate how these responses were distributed across the experiment treatments, determining also any intra-species differences. Data were subjected to general linear model (GLM) analysis to investigate the effect of time, temperature and a_w_ on measured fungal responses. Moreover, effect test was employed pointing the significant effects among treatments and biological responses. Statistical analysis was performed using JMP software (SAS, Institute INV, Cary, NC, USA). The statistical significance was set at *p* ≤ 0.05. Furthermore, two-dimensional (2D) surface response contour plots were generated using Minitab 14 (Minitab Inc., State College, PA, USA) in relation to the combinations of temperature × time for biomass and OTA production for each considered fungus and a_w_ level. Finally, Principal Component Analysis (PCA) was performed on the correlation matrix of the variables as described elsewhere [38]. The explanatory variables used in the analysis were biomass and OTA production, a_w_ levels, incubation temperature and time, fungal strain and the expression of the three selected genes. For the selection of the optimum number of Principal Components (PCs), factors with eigenvalues greater than 1.0 were selected. The results of the PCA analysis were graphically presented by the plots of loadings and scores for the first two principal components indicating a condensed representation of the correlation between the original variables and the distribution of the samples. PCA analysis was implemented with XLStat software version 2006.06 using a (Addinsoft, Paris, France) varimax rotation.

## Figures and Tables

**Table 1:** Analysis of variance for the effect of ecophysiological factors on OTA production.

Data Set	DF	Sum of Squares	F Ratio	Prob > F
Strain (S)	1	102,931.82	152.7185	<0.0001
Temperature (T)	2	137,844.54	102.259	<0.0001
a_w_	1	200,240.91	297.0947	<0.0001
Time (*t*)	2	153,981.37	114.23	<0.0001
T × a_w_	2	88,487.9	65.6441	<0.0001
t × a_w_	2	100,385.72	74.4705	<0.0001
T × t	4	60,879.31	22.5814	<0.0001
T × a_w_ × t	4	43,630.88	16.1836	<0.0001
S × T	2	52,627.61	39.0414	<0.0001
S × a_w_	1	86,572.75	128.4468	<0.0001
S × T × aw	2	45,901.93	34.052	<0.0001
S × T	2	40,530.5	30.0673	<0.0001
S × T × t	4	25,880.57	9.5997	<0.0001
S × T × t × a_w_	4	23,840.98	8.8431	<0.0001
S × t × a_w_	2	39,596.13	29.3741	<0.0001

**Table 2:** Analyses of variance for the effect of ecophysiological factors on: inter-strain differences (**A**); and intra-strain differences (**B**) in transcriptional profiles.

**A.**			
**Genes**	**Dataset**	***p* Value**	**F Ratio**
**AcOTA*nrps***	temperature	0.0541	2.109
(*p* < 0.0103)	a_w_	0.6840	0.0005
	time	0.2600	0.5376
	strain	0.0016 **	10.4009
**AcOTA*pks***	temperature	<0.00021 ***	3.9714
(*p* < 0.0001)	a_w_	0.2339	0.6372
	time	0.0476 *	2.799
	strain	<0.0001***	37.2727
***laeA***	temperature	0.0158 *	3.567
(*p* < 0.0028)	a_w_	0.0368 *	4.1134
	time	0.9761	0.1169
	strain	0.0045 **	8.5874
**B.**			
**Strain**	**Cross Effect**	***p* value**	**F Ratio**
**Ac29**	**AcOTA*nrps***		
	time x temperature	0.0061 **	6.1435
	**AcOTA*pks***		
	time x temperature	0.0011 **	8.7861
	***laeA***		
	time x temperature	0.0047 **	6.5426
	temperature x a_w_	0.0023 **	11.2779
	time x temperature x a_w_	0.0018 **	7.9657
**5010**	**AcOTA*pks***		
	time x temperature x a_w_	<0.0001 ***	13.7201
	***laeA***		
	time x temperarture	<0.0001 ***	20.863
	time x a_w_	0.042 *	4.5396
	time x temperature x a_w_	0.0023 **	7.6165

Significance * *p* < 0.05, ** *p* < 0.001, ** *p* < 0.0001.

**Table 3:** Nucleotide sequences of primers for qPCR assay.

Primer Pair	Gene	Nucleotide Sequences 5′–3′	Reference
F-pks	*AcOTApks*	GTC AAG GTC GGG TGC TAC AA	Lappa et al. (2017)
R-pks		TCG GAA TGA TAC GCG ACT TT	
F-nrps	*AcOTAnrps*	CTC CAC CCA TCC TCC CGT TC	Crespo Sempere et al. (2013)
R-nrps		AAT CCA TGT CCT CAC CAT CGC	
F-laea	*laeA*	CAC CTA TAC AAC CTC CGA ACC AC	Crespo Sempere et al. (2013)
R-laea		GGT TCG GCC AAC CGA CGA CGC TG	
F-tubβ	*β-tubulin*	CGC ATG AAC GTC TAC TTC AAC GAG	Crespo Sempere et al. (2013)
R-tubβ		AGT TGT TAC CAG CAC CGG ACT	

qPCR efficiency tests for each primer were performed under the specific experimental conditions and evaluated as 96% for *AcOTApks*, 101% for *AcOTAnrps*, 98% for *laeA* and 94% for β-*tubulin* genes, respectively.

## Supplementary Materials

The following are available online at www.mdpi.com/2072-6651/9/10/296/s1, Supplementary Figure: Effect of temperature, a_w_ and time on (A) biomass and (B) OTA production of Aspergillus carbonarius strains on SGM (Synthetic Grape Medium). Error bars when visible represent the standard error of the mean value of 3 replicates.
