# Supplementary material for: Monitoring the Temporal Expression of Genes Involved in Ochratoxin A Production of Aspergillus carbonarius under the Influence of Temperature and Water Activity
Source: Toxins (Basel). 2017 Sep 22;9(10):296. doi: 10.3390/toxins9100296 (PMC5666343; doi:10.3390/toxins9100296)
Supplement: Supplementary file 1 [file toxins-09-00296-s001.pdf]

Supplementary Materials: Monitoring the Temporal Expression of Genes Involved in Ochratoxin A Production of Aspergillus carbonarius under the Influence of Temperature and Water Activity.

Iliada K. Lappa, Dimosthenis Kizis and Efstathios Z. Panagou

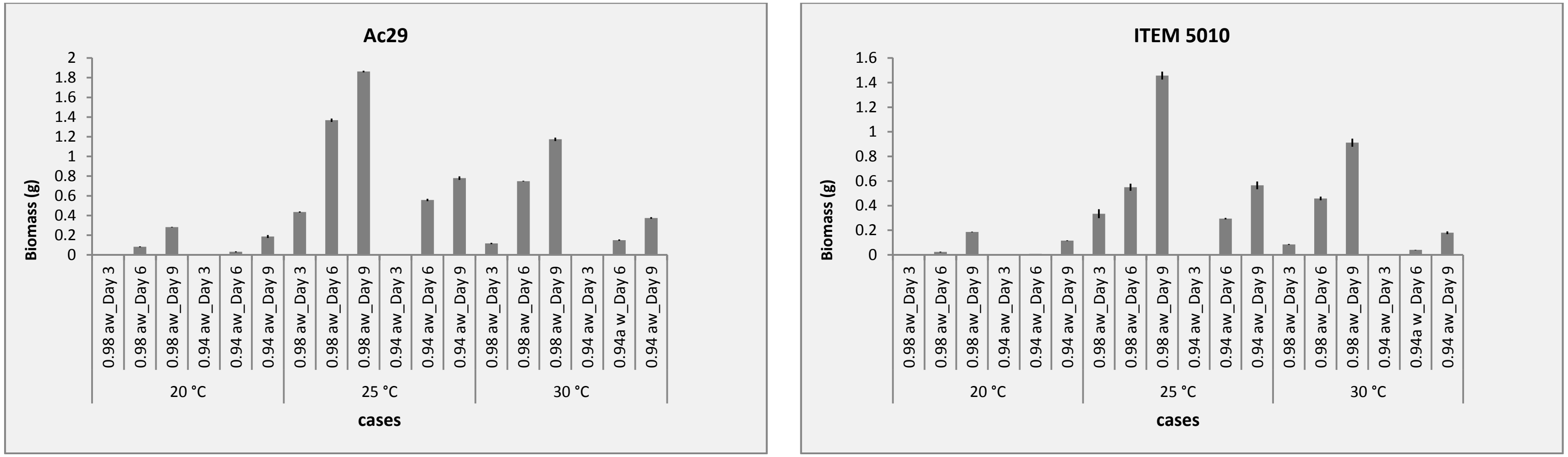

(**A**)

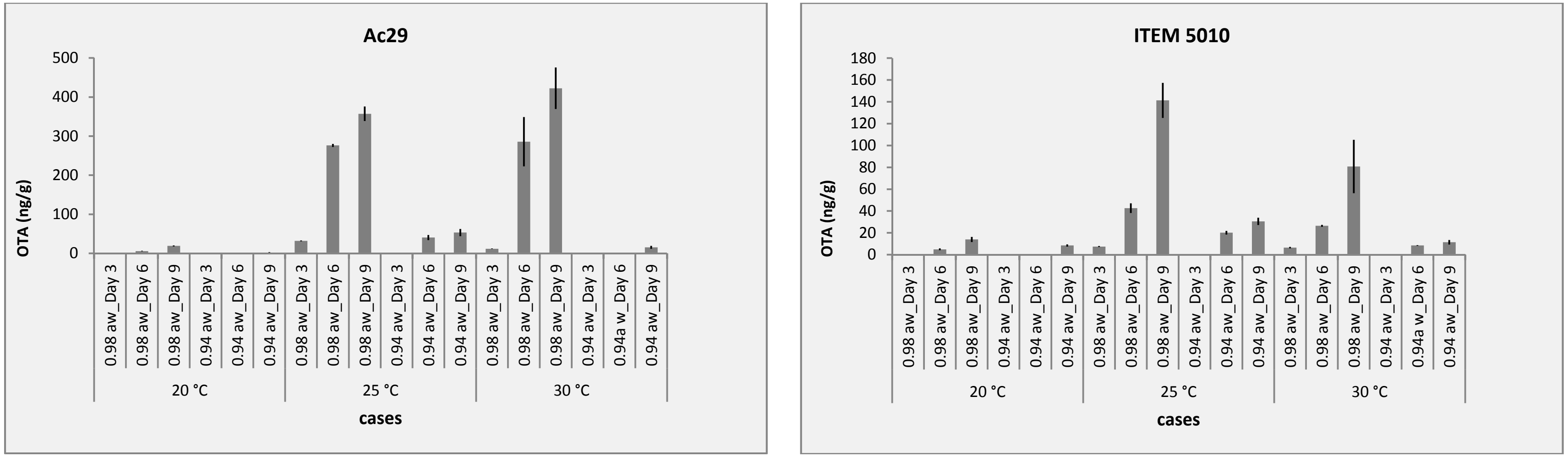

(**B**)

Supplementary Figure: Effect of temperature, a_w and time on (A) biomass and (B) OTA production of *Aspergillus carbonarius* strains on SGM (Synthetic Grape Medium). Error bars when visible represent the standard error of the mean value of 3 replicates.
